# Plasma DNA concentration as an early predictor of outcome in critically ill septic patients

**DOI:** 10.1186/cc9691

**Published:** 2011-03-11

**Authors:** H El-Akabawy, W Radwan, S Gengeehy, A Rezk, A Sisi

**Affiliations:** 1 Cairo University, Cairo, Egypt

## Introduction

Sepsis is associated with cell necrosis and apoptosis. Indeed, plasma DNA levels have been shown to be increased in patients with sepsis [1]. So we investigated the prognostic value of circulating levels of cell-free DNA in critically ill septic patients regarding the clinical course and final outcome.

## Methods

A total of 80 critically ill septic patients were included in a prospective, randomized, single-center study. All were subjected to the measurement of cell-free plasma DNA concentrations (by real-time PCR assay for the β-globin gene), CRP levels and procalcitonin concentrations, all measured on ICU admission. APACHE II and SOFA scores were calculated. Clinical outcome (duration of ICU stay, need for MV, need for inotropic/vasopressor support, need for haemodialysis, and final outcome of survival/mortality rates) were recorded for all patients.

## Results

The median plasma DNA concentration in critically ill septic patients was 195.7 ng/ml and this was significantly (approximately sevenfold) higher than the DNA concentration in healthy subjects 27 ng/ml (*P *< 0.001). The median DNA concentration was significantly higher in those who need MV (205.6 ng/ml vs. 123.7 ng/ml; *P *= 0.006), in those who were on inotropic/vasopressor support (234.6 ng/ml vs. 114.7 ng/ml; *P *< 0.001) and in those who required renal supportive therapy (haemodialysis) (244.2 ng/ml vs. 181.1 ng/ml; *P *= 0.001). DNA concentration demonstrated a highly significant correlation with CRP concentration (*r *= 0.661, *P *< 0.001), procalcitonin concentration (*r *= 0.820, *P *< 0.001), SOFA score (*r *= 0.710, *P *< 0.001), and APACHE II score (*r *= 0.559, *P *< 0.001). The median plasma DNA concentration in nonsurvivors (38 of 80 patients, 47.5%) was 234.8 ng/ml, and this was significantly (approximately twofold) higher than that in survivors (115.5 ng/ml, *P *< 0.001). Receiver operator characteristic analysis of the data indicated a sensitivity of 95% and a specificity of 81% when DNA concentration of 186.5 ng/ml was taken as a predictor of ICU mortality.

## Conclusions

Plasma cell-free DNA may be a potentially useful marker for the evaluation of ICU septic patients and for the prediction of their adverse outcomes. The ability for rapid risk stratification may allow clinicians to make more rational therapeutic decisions to ensure that the hospital resources are used efficiently and appropriately.
